# Global Analysis of Dark- and Heat-Regulated Alternative Splicing in Arabidopsis

**DOI:** 10.3390/ijms24065299

**Published:** 2023-03-10

**Authors:** Di Zhang, Mo-Xian Chen, Mehtab Muhammad Aslam, Ying-Gao Liu, Jianhua Zhang

**Affiliations:** 1 National Key Laboratory of Green Pesticide, Key Laboratory of Green Pesticide and Agricultural Bioengineering, Ministry of Education, Center for R&D of Fine Chemicals of Guizhou University, Guiyang 550025, China; 2 Department of Biology, Hong Kong Baptist University, and State Key Laboratory of Agrobiotechnology, The Chinese University of Hong Kong, Shatin, Hong Kong; 3 State Key Laboratory of Crop Biology, College of Life Science, Shandong Agricultural University, Taian 271018, China

**Keywords:** alternative splicing, dark, heat stress, abiotic stresses, splicing-related genes, splicing factors, *SR30*, RNA sequencing

## Abstract

Alternative splicing (AS) is one of the major post-transcriptional regulation mechanisms that contributes to plant responses to various environmental perturbations. Darkness and heat are two common abiotic factors affecting plant growth, yet the involvement and regulation of AS in the plant responses to these signals remain insufficiently examined. In this study, we subjected Arabidopsis seedlings to 6 h of darkness or heat stress and analyzed their transcriptome through short-read RNA sequencing. We revealed that both treatments altered the transcription and AS of a subset of genes yet with different mechanisms. Dark-regulated AS events were found enriched in photosynthesis and light signaling pathways, while heat-regulated AS events were enriched in responses to abiotic stresses but not in heat-responsive genes, which responded primarily through transcriptional regulation. The AS of splicing-related genes (SRGs) was susceptible to both treatments; while dark treatment mostly regulated the AS of these genes, heat had a strong effect on both their transcription and AS. PCR analysis showed that the AS of the Serine/Arginine-rich family gene *SR30* was reversely regulated by dark and heat, and heat induced the upregulation of multiple minor *SR30* isoforms with intron retention. Our results suggest that AS participates in plant responses to these two abiotic signals and reveal the regulation of splicing regulators during these processes.

## 1. Introduction

Plants are sessile organisms, and, thus, adapting themselves to the ever-changing environment is crucial for their survival and reproduction. In the last two decades, an increasing number of studies have revealed the contribution of alternative splicing (AS) in plant responses to various environmental stimuli and stresses [1]. In higher eukaryotes, a single pre-mRNA can be processed into multiple mRNA transcripts by AS. While a portion of these mRNA isoforms generated by AS can be further translated into various protein isoforms [2,3], some other RNA isoforms contain premature termination codons (PTCs) and are degraded by the nonsense-mediated RNA decay (NMD) pathway, a mechanism that controls protein level [4,5]. As a result, AS greatly enhances the coding capacity and versatility of eukaryotic genomes. In Arabidopsis, it has been estimated that 61% of the intron-containing genes are alternatively spliced [6], and many transcriptome studies have revealed that changes in the global AS landscape are part of the plant responses to environmental stresses or signals [7]. Meanwhile, the biological functions of many stress-related AS events have been characterized. For example, group A protein type 2C phosphatase gene *HAB1* has been reported to fine-tune abscisic acid (ABA) signaling by producing two splice isoforms with opposite functions [8]. Therefore, characterization and understanding of the changes in plant AS profile might provide insight into the mechanisms of plant adaption to various stresses.

The molecular machinery that conducts mRNA splicing is a dynamic complex known as the spliceosome, which consists of five uridine-rich small nuclear ribonucleoprotein particles (U snRNPs), U1, U2, U5, and U4/6, together with numerous non-snRNP protein factors [9]. The spliceosomal components recognize the consensus sequences located at the two ends of introns, which typically harbor highly conserved dinucleotides, 5′-GT-AG-3′, and perform splicing through two catalytic reactions [9]. In higher eucaryotes, the selection of the splices sites by the spliceosome is highly regulated by the interaction between cis-elements in the pre-mRNAs and regulatory proteins such as Serine/Arginine-rich (SR) proteins and heterogeneous nuclear ribonucleoprotein particle (hnRNP) proteins [10]. It has been hypothesized that stress-specific AS events are controlled by altered amounts of splicing factors under different conditions [11]. Intriguingly, splicing regulator genes themselves also undergo extensive AS and display tissue- or stress-specific AS events, such as those among SR genes [12], which likely adds to the complexity of AS regulation in plants. Meanwhile, stress-responsive AS events are often found enriched in splicing factor genes [13,14], and Shikata et al. reported that splicing factor genes are the main early targets of AS control in the phytochrome signaling pathway [15]. Therefore, understanding the regulation of splicing regulator genes is important for understanding the regulation of AS in plant responses to environmental stresses.

Light and temperature are two key environmental factors affecting plant growth and development [16]. Light is vital to most aspects of plant growth, and exposure to darkness is known to suppress photosynthesis, cause carbon starvation, and induce etiolation and senescence [17,18,19]. A number of dark-inducible (*DIN*) genes have been identified, including early-responsive *DIN* genes and late-responsive *DIN* genes, depending on the length of dark exposure required for induction [20,21]. However, compared to the well-reviewed light signaling pathways, knowledge about the plant dark response is relatively limited. It has been reported that light intensively regulates AS [22,23,24], which suggests the involvement of AS in the plant dark responses. Compared to darkness, heat stress is a more intense stress that causes harm such as photosynthesis inhibition, membrane dysfunction, oxidative damage, and dehydration, thereby severely disrupting plant growth [25]. Particularly, due to global warming, high temperature has become one of the major abiotic stresses affecting crop yield [26], and, thus, understanding the mechanisms of plant heat responses has become increasingly necessary. Thus far, the transcriptional regulation of plant heat responses has been well-characterized, which involves activation of a series of heat shock factors and heat shock proteins, which facilitate protein folding and reduce the negative effects caused by the heat stress [27]. Recent reports have shown that AS also participates in the plant heat response and functions in heat shock memory [28,29]. However, the regulation of the plant AS responses and the role of splicing factors during plant heat responses remain insufficiently studied. Therefore, to gain insight into the plant responses to these two abiotic signals, we measured here the effects of darkness and heat stress on global AS events, especially those among splicing-related genes (SRGs), in the model species *Arabidopsis thaliana*. We applied these two treatments together in one study, partially for the convenience in the conduction of the experiments and partially for the purpose of comparing these different abiotic signals. Our results indicated that both darkness and heat were involved in the regulation of a large amount of substrate genes, including SRGs, and showed the different regulation patterns between these two treatments.

## 2. Results

### 2.1. Identification of Dark- and Heat-Regulated DEGs and DAS Events

**Figure 1:**
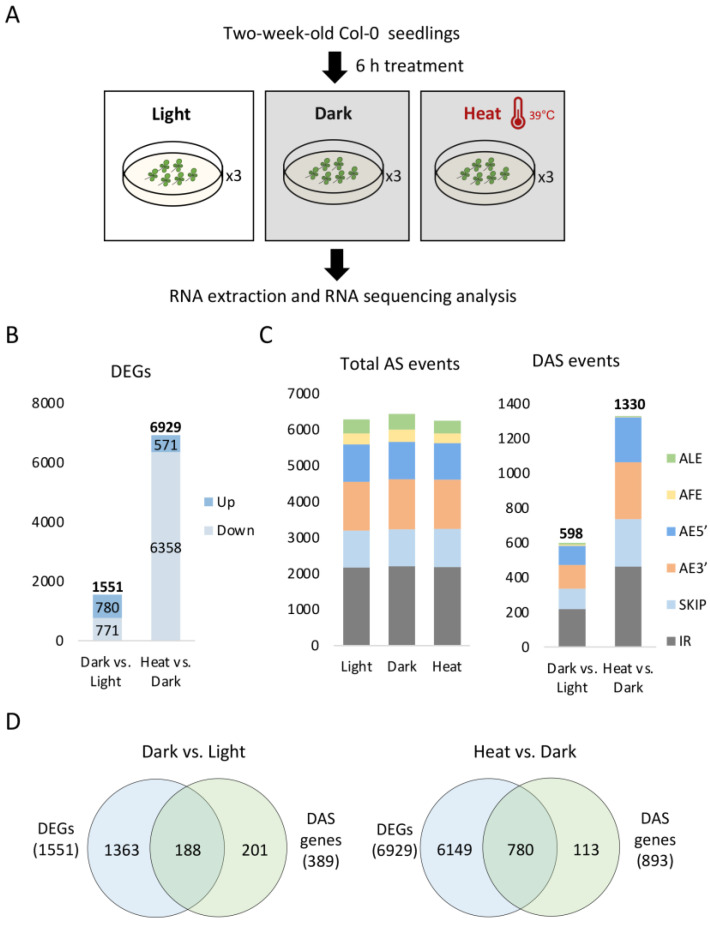
DEGs and DAS events identified upon dark or heat treatments. (**A**) Schematic view of the dark and heat treatments conducted for transcriptome analysis. Each treatment had three biological replicates. (**B**) Numbers of identified DEGs. (**C**) Different AS types of total AS events and DAS events. (**D**) Venn diagrams comparing DEGs and DAS genes.

To study the global effects of darkness and heat stress on the AS of plants, two-week-old light-grown Arabidopsis seedlings were subjected to darkness or heat stress treatments for 6 h or kept unchanged at their original condition with regular light exposure (Figure 1A). The heat-treated seedlings were also kept in darkness to avoid the introduction of photooxidative stress [30,31]. Seedlings placed under normal light conditions served as the control for dark-treated seedlings, while dark-treated seedlings served as the control for heat-treated seedlings. After the treatments, all samples were subjected to RNA sequencing. Approximately 60 million clean reads per sample were obtained and ~97% of them were uniquely mapped to the Arabidopsis reference genome (Araport11), and the resulting reads were used for subsequent analysis (Table S1).

Differentially expressed genes (DEGs) were identified by the comparison of corresponding transcriptomes as mentioned above (Dataset S1). In total, dark and heat treatments caused 1551 and 6929 DEGs, respectively (Figure 1B). Upon dark treatment, roughly the same number of genes, i.e., 780 and 771, were upregulated and downregulated, respectively, whilst the number of genes downregulated by heat (6358) was ten times higher than that of the genes upregulated by heat (571). To validate the effectiveness of the dark and heat treatments in our study, DEGs identified in our dataset were compared with previously reported dark-responsive genes [19,21,32,33] and heat-responsive genes [34,35,36,37,38,39,40,41,42,43,44,45,46,47] (Figure S1; Dataset S3A,B). We found that 17 out of the 30 previously reported dark-responsive genes were similarly regulated in this study and 55 out of the 80 heat-responsive genes (mostly heat shock factors and heat shock proteins) were also similarly upregulated in this study. Therefore, the treatments in our study were effective. 

Total AS events and differentially expressed AS (DAS) events were both identified (Figure 1C; Dataset S1). More than 6000 AS events were identified in each sample, including alternative 5′ splice site (AE5′), alternative 3′ splice site (AE3′), intron retention (IR), exon skipping (SKIP) or cassette exon inclusion (CE), alternative first exon (AFE), and alternative last exon (ALE). Dark and heat treatment each caused 598 and 1330 DAS events, affecting 389 and 893 genes (each gene had about 1.5 DAS events on average), respectively, suggesting that the two treatments both had a widespread impact on the global AS patterns. Among the six AS types, IR was the most abundant event among both total AS events and DAS events, which is similar as previously reported [48]. We also analyzed and compared the splice sites associated with the identified total AS events and DAS events (Figure S2). In our study, the occurrence rate of the classic GT and AG dinucleotides at the splice sites was around 80%. The heat treatment increased the usage of these two conventional dinucleotides and reduced the usage of non-canonical sites, while the dark treatment reduced the usage of GT and AG but to a lesser extent, suggesting that the two treatments both affected splice sites selection, yet with opposite effects.

### 2.2. Comparison of GO Terms Enriched in DEGs and DAS Genes

**Figure 2:**
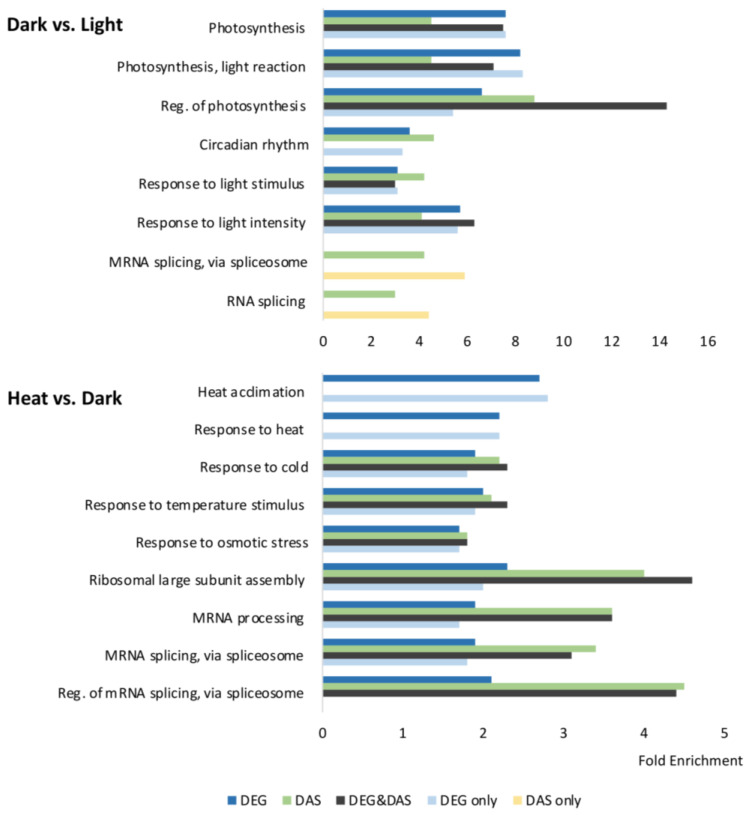
GO analysis of dark- or heat-regulated DEGs and DAS genes. Blue, green, gray, cyan, and yellow bars represent the fold enrichment of total DEGs, total DAS genes, overlapping genes between DEG and DAS genes, DEGs only, and DAS genes only, respectively, corresponding to those shown in the Venn diagrams in Figure 1D.

We then analyzed the overlap between DEGs and DAS genes (Figure 1D) as well as their enriched gene ontology (GO) terms under dark and heat treatments (Figure 2; Figure S3; Dataset S2). There were significantly more identified DEGs than DAS genes under both conditions. Upon dark treatment, approximately half of the DAS genes overlapped with DEG genes, suggesting that these genes were co-regulated via transcription and AS by the dark signal. Meanwhile, the remaining half of the DAS genes were specific to AS regulation, suggesting that AS had an independent role in the plant responses to dark treatment apart from transcriptional regulation. The GO terms for DEG-only genes and shared genes between DEGs and DAS genes were mostly enriched in light-related biological processes such as photosynthesis, the response to light intensity, and the circadian rhythm, indicating that these processes were regulated by both transcriptional regulation and AS. By contrast, DAS-only genes were barely found in those groups but were greatly enriched in mRNA splicing, in which the enrichment of DEGs was not found, suggesting that the splicing machinery itself was regulated primarily at the level of AS by the dark signal.

Upon heat treatment, most of the DAS genes overlapped with DEG genes (Figure 1D). As shown in Figure 2 and Figure S3, heat-regulated DEGs were enriched in heat acclimation, responses to heat, and mRNA splicing. Meanwhile, heat-regulated DAS genes were enriched in responses to temperature stimuli and abiotic stresses (e.g., cold and osmotic stresses), but were barely found in response to heat stress. Instead, heat-regulated DAS genes were preferentially enriched in mRNA splicing, mRNA processing, and the circadian rhythm (Figure S3), suggesting that the AS responses during plant heat stress responses mostly involve the mRNA splicing machinery. The observation that mRNA splicing-related GO terms were enriched by both DEGs and DAS genes indicates that heat was able to affect both the transcription and AS of SRGs. In addition, only about 13% of the heat-regulated DAS genes were only affected by AS and they were enriched in the circadian rhythm and light signaling pathways (Dataset S2).

### 2.3. Analysis of Dark- and Heat-Regulated DAS Events by qPCR

**Figure 3:**
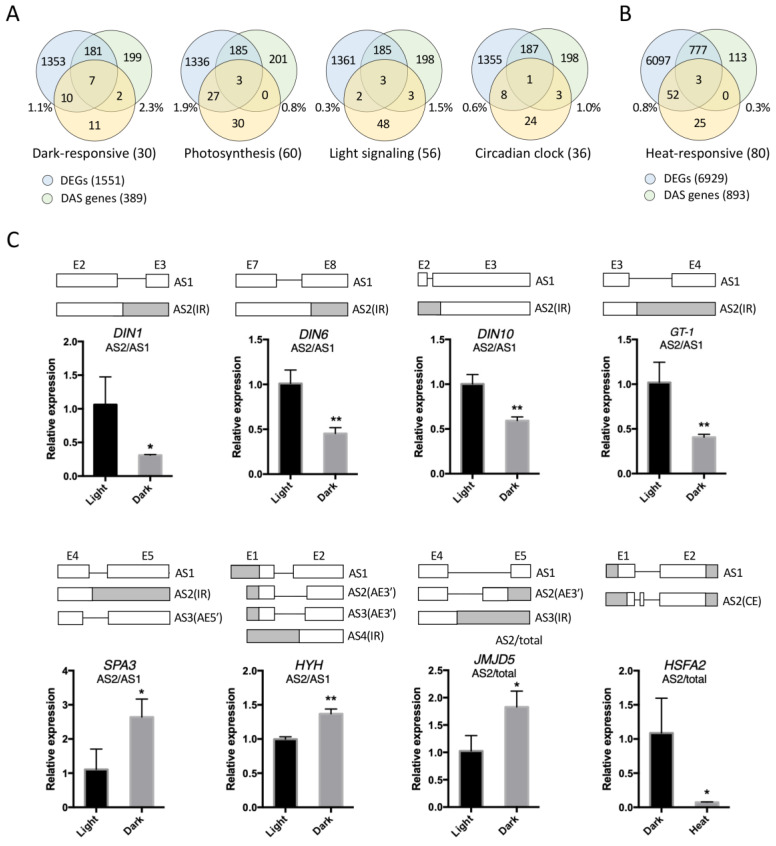
qPCR analysis of dark- and heat-regulated AS events. Venn diagrams comparing DEGs and DAS genes regulated by dark (**A**) or heat (**B**) treatments with genes from different functional categories. Percentages indicate the ratio of enriched genes in each functional group relative to the total DEGs or DAS genes. (**C**) qPCR analysis of dark- or heat-regulated AS events of representative genes from different functional categories. Partial gene models illustrate the alternatively spliced regions. White and gray boxes indicate CDS and untranslated region, respectively. Numbers indicate the order of the exons. The ratios of the amount of one of the alternative transcripts to that of the primary transcript or total transcripts are shown in the bar charts. Error bars represent SE from three biological replicates. Asterisks indicate significant differences (*, *p* < 0.05; **, *p* < 0.01; Student’s *t*-test).

To further understand the physiological roles of identified DEGs and DAS genes, we compared them with a number of our manually generated gene lists, which contain genes that have been reported to be involved in dark responses, photosynthesis [49,50,51,52,53,54,55,56,57,58,59,60], light signaling [33,51,61,62,63,64,65,66,67,68,69,70,71,72,73,74,75,76,77,78,79,80], the circadian clock [77,81,82,83,84,85,86,87,88,89,90,91,92,93,94,95,96], and heat stress responses (Figure 3A,B; Dataset S3). Exactly half of the photosynthesis genes in our list overlapped with dark-regulated DEGs and DAS genes, and the overlapping genes were mostly enriched in DEGs. Genes from the other three categories all showed a high enrichment factor (indicated by the percentage near the corresponding circle) in DAS genes than DEGs, implying that AS might play a more important role in these three pathways compared to photosynthesis. Particularly, there were more genes in the light signaling group that overlapped with DAS genes than DEGs, despite the total number of DEGs being much higher than those of DAS genes. Upon heat treatment, the heat-responsive genes were enriched much more in DEGs than DAS genes, which is consistent with our GO enrichment results in Figure 2, indicating that the regulation of heat-responsive genes primarily depends on transcriptional regulation.

We then selected representative DAS genes from each functional category and analyzed their AS pattern by quantitative real-time PCR (qPCR) analysis. Specifically, we measured the ratio of the amount of an alternative splice transcript to that of the primary transcript or total transcripts. Among the 11 genes we tested in total, 8 (about 73%) of them showed significant changes in their AS pattern (Figure 3C). These 8 genes included dark-inducible (*DIN*) genes (*DIN1*, *DIN6,* and *DIN10*), a regulatory gene of photosynthesis (*GT-1*), light signaling components (*SPA3* and *HYH*), a circadian clock-related gene (*JMJD5*), and a heat shock factor gene (*HSFA2*), further proving that dark and heat treatments in our study were able to regulate the AS of downstream genes. Particularly, the IR events of the three tested *DIN* genes, *DIN1*, *DIN6* and *DIN10*, all reduced in dark treatment, which means that the percentage of their primary transcripts encoding the full-length protein increased, suggesting that the induction of these genes by dark treatment did not only occur at the transcriptional level, but also at the post-transcription level via AS. 

### 2.4. DAS Genes Co-Regulated by Dark and Heat Treatments Were Enriched in mRNA Splicing

**Figure 4:**
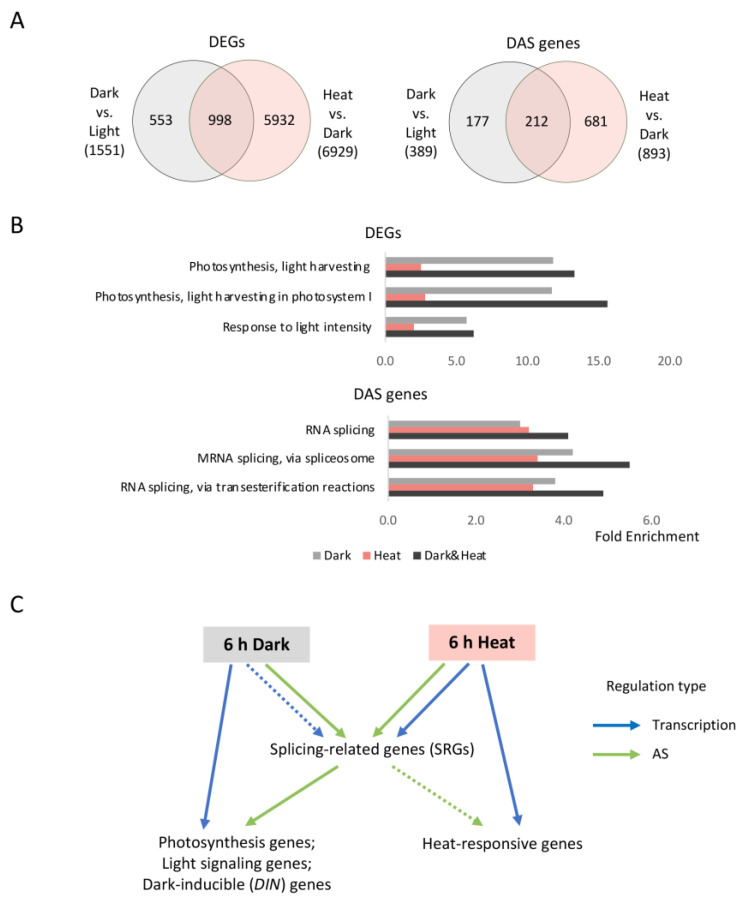
DAS genes co-regulated by dark and heat treatments were enriched in mRNA splicing. (**A**) Venn diagrams showing genes that were co-regulated by dark and heat treatments. (**B**) Representative GO terms of DEGs and DAS genes that were co-regulated by dark and heat treatments. (**C**) A proposed model illustrating the regulation of dark and heat treatments on the transcriptome and AS in Arabidopsis. Blue arrows indicate transcriptional regulation and green arrows indicate regulation via AS. Arrows drawn with solid lines represent a major regulation type, while those drawn with dotted lines indicate a minor regulation type in the corresponding pathways.

To study the overlap between plant responses to dark and heat treatments, we compared dark-regulated genes and heat-regulated genes in Venn diagrams. Approximately two-thirds of the dark-regulated DEGs (998 out of 1551) overlapped with those regulated by the heat treatment (Figure 4A), and these 998 genes were mostly enriched in photosynthesis (Figure 4B; Figure S4; Dataset S2), which is consistent with current knowledge that both darkness and heat inhibit photosynthesis. Meanwhile, 212 dark-regulated DAS genes overlapped with those regulated by heat (Figure 4A), and these genes were mainly enriched in splicing-related GO terms (Figure 4B; Figure S4; Dataset S2). Our previous GO analysis (Figure 2) showed that both dark- and heat-regulated DAS genes were enriched in mRNA splicing, suggesting that SRGs themselves are extensively regulated by AS. Here, we further revealed that at least part of those SRGs were co-regulated by dark and heat, suggesting that these genes might lie at the crosstalk between AS regulation pathways induced by dark and heat treatments. Based on the results in Figure 2 and here, we proposed a model illustrating the regulation of dark and heat treatments on the transcriptome and AS in Arabidopsis (Figure 4C). 

### 2.5. Identification and Analysis of Dark- and Heat-Regulated DEGs and DAS Events among SRGs

**Figure 5:**
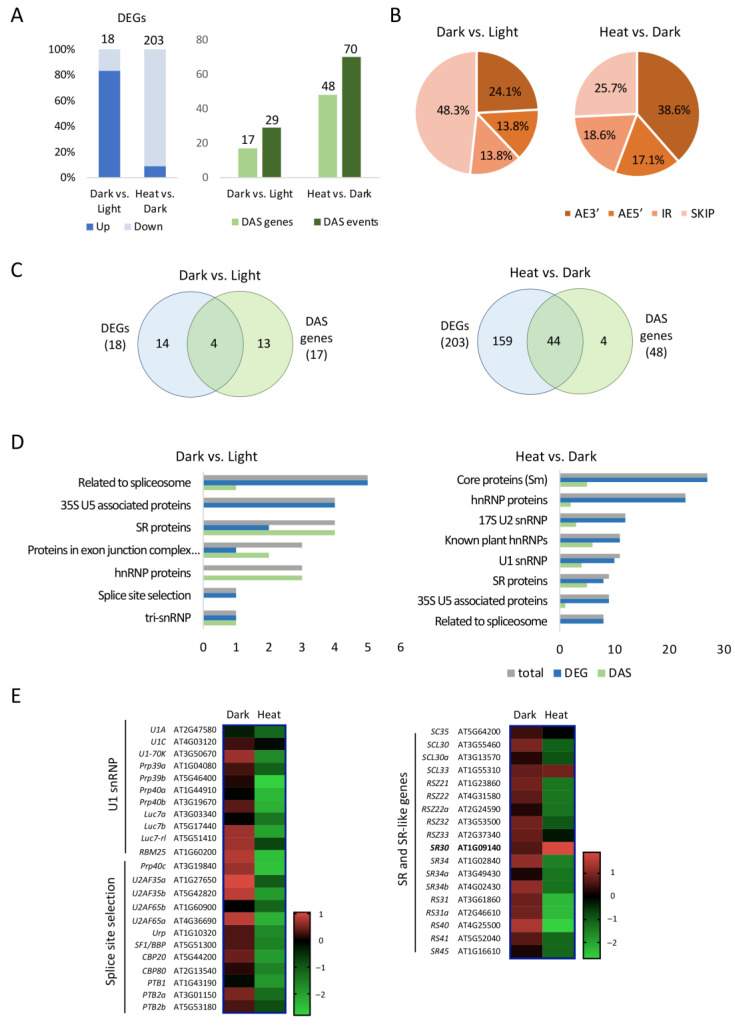
Analysis of SRGs that are regulated by dark and heat treatments. (**A**) Numbers of DEGs, DAS genes, and DAS events. (**B**) Pie charts illustrating different AS types of the DAS events identified. (**C**) Venn diagrams comparing DEGs and DAS genes. (**D**) Subgroup classification of DEGs and DAS genes. (**E**) Heat maps showing expression profile of genes from selected subgroups. Red and green colors indicate up-regulation and down-regulation, respectively. Numbers indicate fold change. *SR30* is highlighted in bold.

Next, to gain insight into the mechanism of AS regulation upon dark and heat treatments, we studied how SRGs themselves were regulated. In Arabidopsis, there are more than 400 genes that have been identified as SRGs (PlantSPEAD; http://chemyang.ccnu.edu.cn/ccb/database/PlantSPEAD/; accessed on 1 May 2022). By comparing these genes (Dataset S3F) with our own dataset, we identified splicing-related DEGs and DAS events under dark and heat treatments (Figure 5). 

Dark treatment altered the transcription and AS of 18 and 17 SRGs, respectively (Figure 5A). These 17 DAS genes showed 29 DAS events, and nearly half of the DAS events were exon skipping, while AE3′, AE5′, and IR made up the remaining half of the total events (Figure 5B). DEGs and DAS genes exhibited limited overlapping among SRGs (Figure 5C), suggesting that they were regulated by different mechanisms. The 31 dark-regulated SRGs were classified into different subgroups based on the classification of the Splicing-Related Gene Database (SRGD; http://www.plantgdb.org/SRGD/ASRG/ASRP-home.php; accessed on 1 May 2022), including different types of constitutive members of the spliceosome and regulatory proteins such as SR proteins and hnRNP proteins (Figure 5D). While DEGs were preferentially enriched in groups consisting of constitutive spliceosomal proteins, DAS genes were preferentially enriched in regulatory protein categories. Specifically, SR proteins and hnRNP proteins both contained more DAS genes than DEGs, suggesting that these splicing regulators were regulated mostly by AS during dark treatment. 

Heat caused 203 DEGs and 48 DAS genes among SRGs, and the majority of DAS genes overlapped with DEGs (Figure 5A). The distribution of different AS types among DAS events, with AE3′ being the most abundant type, was different from those induced by the dark. Heat-regulated SRGs also covered different subgroups, such as spliceosomal core proteins, hnRNP proteins, and SR proteins (Figure 5D). While DAS genes were mostly enriched in regulatory proteins, DEGs were abundantly enriched in both core proteins and hnRNP proteins, indicating that heat may regulate global AS by affecting both the transcriptional level and AS of splicing regulators. 

We also noticed that most of the dark-regulated DEGs were upregulated while most of the heat-regulated DEGs were downregulated (Figure 5A). Expression data of genes from three representative subgroups of SRGs: U1 snRNP, Splice site selection, and SR or SR-like proteins are presented in Figure 5E. Most of these SRGs were slightly upregulated by the dark while they were greatly suppressed under heat. The different regulation of the transcription and AS of SRGs likely caused the different global AS pattern under these two treatments. Interestingly, two SR genes, *SR30* and *SCL33*, especially *SR30*, showed a distinctive upregulation upon heat stress. The upregulation of *SR30* was strongest among all SR genes, while the three homologs of *SR30* from the same SR subfamily (i.e., *SR34*, *SR34a,* and *SR34b*) all showed a similar degree of slight downregulation, implying that *SR30* might play a unique role during plant heat response.

### 2.6. Analysis of Dark- and Heat-Regulated DAS Events among SRGs by qPCR and RT-PCR 

**Figure 6:**
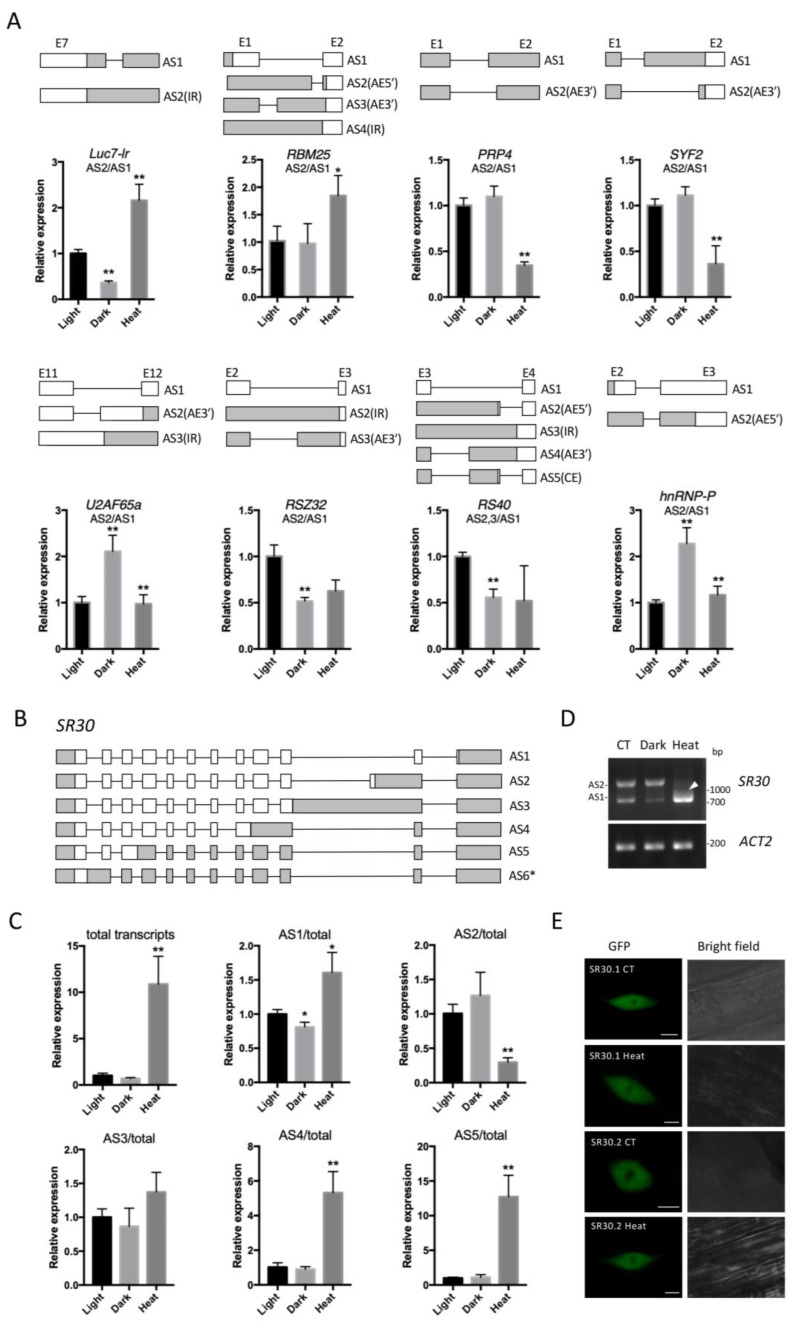
qPCR analysis of dark- or heat-regulated AS events among SRGs. (**A**) qPCR analysis of the AS events of SRGs from different subgroups, similar to that described in Figure 3C. Specifically, a significant difference was calculated using expression data between dark and light or between heat and dark groups. Error bars represent SE from three biological replicates. Asterisks indicate significant differences (*, *p* < 0.05; **, *p* < 0.01; Student’s *t*-test). (**B**) Schematic view of *SR30* splice isoforms. White and gray boxes indicate CDS and untranslated region, respectively. Asterisk indicates a novel transcript identified in this study. (**C**) The relative expression of *SR30* relative to *ACT2* or the ratios of the amount of individual *SR30* isoforms to that of total transcripts was analyzed by qPCR. (**D**) *SR30* was detected using a pair of primers targeting the CDS of AS1 by RT-PCR. The two major bands represent AS1 and AS2. The white triangle indicates a smear region between AS1 and AS2. (**E**) Subcellular localization of SR30.1 and SR30.2 protein isoforms in transgenic Arabidopsis with or without 6 h of heat treatment. CT, control. Bars = 5 μm.

We then analyzed the AS of representative DAS genes from different splicing subgroups, including U1 snRNP (*Luc7-lr* and *RBM25*), U4/6 snRNP (*PRP4*), U5-associated protein (*SYF2*), Splice site Selection (*U2AF65a*), SR protein (*RSZ32* and *RS40*), and hnRNP protein (*hnRNP-P*), by qPCR (Figure 6A). For genes with multiple transcript isoforms, we selectively tested some of the AS events. The significant changes detected by qPCR further showed that the AS of SRGs was regulated by the corresponding treatment. Particularly, the AS in *Luc7-lr*, *U2AF65a,* and *hnRNP-P* responded to both treatments, suggesting that the AS of these genes was susceptible to different abiotic signals. 

As our results indicated that *SR30* was strongly upregulated by heat stress among SRGs (Figure 5E), we measured the transcription and AS of *SR30* by both qPCR and reverse-transcription (RT)-PCR (Figure 6C,D). The SR genes are famous for undergoing extensive AS, and multiple splice isoforms of *SR30* have been reported by previous publications [12,97,98], which are illustrated in Figure 6B. Among them, AS1, AS2, and AS3 correspond to AT1G09140.1, AT1G09140.2, and AT1G09140.3, annotated by the Araport11 database. Particularly, AS1 and AS2, which are caused by one AE3′ event that occurred in the tenth intron, are the most abundant isoforms, but only AS1 encodes for the full-length SR30 protein, which can be detected by antibodies in a phosphorylated status in the wide-type Arabidopsis [97]. Compared to *SR30.1*, *SR30.2* associates with ribosomes more weakly and was also detected less when overexpressed in transgenic Arabidopsis [99], suggesting that it might be less functional at the protein level. In the RT-PCR assay, we detected both AS1 and AS2 using one pair of primer targeting the coding sequence (CDS) of AS1 (Figure 6D). Interestingly, the two isoforms exhibited an opposite regulation pattern under dark and heat treatments (Figure 6C,D). Namely, upon dark treatment, AS1 reduced and AS2 became much more abundant, while upon heat treatment, AS2 reduced and AS1 became the major isoform. This observation is consistent with the previously reported results [98,99]. The increase in the ratio of AS1 suggests that more functional proteins translated from AS1 will be produced. By contrast, AS3, in which the alternatively spliced tenth intron is fully retained, remained stable upon both treatments (Figure 6C). Surprisingly, AS4 and AS5, which also contained intron retention, both showed drastic upregulation under heat treatment (Figure 6C). Accordingly, a smear area (indicated by a white arow) occurred between AS1 and AS2 on the DNA gel figure (Figure 6D), suggesting the emergence or upregulation of minor *SR30* isoforms other than AS1 and AS2. DNA fragments were then extracted from the smear area and subjected to Sanger sequencing. While most of the identified fragments turned out to be AS5, we also identified a novel isoform, AS6, which had an intron retention in the first intron of *SR30* (Figure S5). The retained introns in AS4, AS5, and AS6 were all much shorter than the one in AS3, implying that the short introns of *SR30* are more likely to be retained under heat stress. Our results indicate that heat can massively affect the expression and AS of *SR30*. 

We also examined the subcellular localization pattern of the protein products generated by the CDS of *SR30* AS1 (i.e., SR30.1) and *SR30* AS2 (i.e., SR30.2). Both SR30.1 and SR30.2 proteins were distributed in the cell nucleus and were slightly excluded from the nucleolus (Figure 6E). After 6 h of heat treatment, their localization pattern did not show significant changes compared to the control, suggesting that their nuclear distribution was relatively stable under heat conditions.

## 3. Discussion

In this study, we analyzed the AS profiles of Arabidopsis seedlings upon dark and heat treatments and revealed that each treatment affected the AS of a subset of genes. Here, the plants were subjected to a short-term dark treatment for 6 h, which may provide information on the initial molecular changes that occur in plants exposed to darkness. We discovered that our dark treatment altered the AS of many *DIN* genes (Figure 3A,C) such as *DIN1*, *DIN6,* and *DIN10*, which are known early-responsive *DIN* genes that start to accumulate within 3 h of exposure to darkness and play roles in plant responses to sugar starvation and leaf senescence [21], suggesting that AS might contribute to at least the early stage of plant adaptation to darkness. Meanwhile, dark-regulated AS events were also found enriched in a variety of functional categories related to light-related processes, such as photosynthesis, light signaling, and the circadian clock (Figure 2 and Figure 3). The AS of genes from these functional categories has also been reported to be regulated by light exposure [15,100]. Therefore, our findings further provide evidence that AS might be extensively involved in plant responses to different light conditions or the light/dark transition. Under heat stress, we found that heat-regulated AS events were enriched in responses to temperature stimuli and abiotic stresses, suggesting that AS might regulate stress-related genes and contribute to plant adaptation to heat stress. In this study, we applied heat treatment in the dark to avoid introducing additional stress factors; in nature, however, plants are more commonly exposed to heat stress in the light. Our data also showed that dark treatment changed the AS of many SRGs, suggesting that the AS profile during plant heat stress responses in the light might differ from that in the dark, which could also be examined in the future to gain more insight into the role of AS in plant heat stress adaptation. 

We also compared the role of transcriptional regulation and AS regulation during plants responses to these two abiotic signals, as summarized in Figure 4C. Our results indicated that dark and heat signals each preferentially triggered different regulation mechanisms with different substrate genes, possibly for the realization of maximum regulation efficiency in different conditions. Particularly, the AS of SRGs was regulated by both treatments. Considering that SRGs, especially splicing regulator genes, tend to undergo extensive AS [12], it is likely that they are more sensitive to AS regulation than transcriptional regulation under different conditions. Our results are consistent with previous reports that also showed the enrichment of RNA-binding proteins in light-responsive AS events [15,101] and heat-responsive AS events [28,29]. Interestingly, Kannan et al. reported that the GO term RNA processing was enriched in heat-responsive AS events in Arabidopsis but not in its thermotolerant relative *Boechera depauperata* [29], implying that alteration in the AS of SRGs might be involved in the heat tolerance of plants. In addition, the different regulation mechanisms exhibited by our dark and heat treatments might be caused by the intrinsic difference of these two types of signals. For example, in our study, 6 h of darkness was a mild unfavorable condition, while 6 h of heat was a lot more threatening to the survival and growth of plants; thus, it is likely that the response of splicing factor genes to mild stress signals is preferential via a change in their AS but undergo transcriptional change in response to severe stresses. 

Upon heat treatment in our study, a counterintuitive observation is that DAS events were barely found in the GO terms of heat responses or heat acclimation, while they were enriched in relative functional groups such as responses to cold and responses to osmotic stress. A number of heat shock factors and heat shock proteins that undergo AS have been reported and the most studied gene is *HSFA2*, which is an important member of heat shock response and exhibits heat-induced splice variants [102,103]. In our study, we also showed that its AS was altered by heat stress (Figure 3C). However, our RNA sequencing analysis pipeline hardly detected DAS in most other HSFs. This phenomenon may indicate that heat-responsive genes are, in general, less regulated via AS compared to transcriptional regulation. On the other hand, the identification of AS in our study was based on the annotation of the Araport11 database. Even though it is the latest release of Arabidopsis genome annotation, it appears that the AS of many heat-responsive genes is still under-annotated. It has been reported that heat caused the splicing repression and upregulation of IR, especially in heat-response genes [28]. In our study, we found that SRGs tended to be downregulated in general by heat stress (Figure 5A,E), which likely contributed to the global splicing repression and reduction in the usage of non-canonical splice sites (Figure S2). IR was also found to be the most abundant DAS events during heat stress in our study, though we did not observe a drastic increase in the number of IR, probably reflecting the limitation of our analysis. Moreover, it has been recently reported that IRs are not reliably detected by short RNA-sequencing reads compared to long RNA-sequencing reads [104]. In the future, to detect more novel transcripts, a combined assembly strategy that merges a reference-based assembly and de novo transcriptome assembly can also be applied to create a more comprehensive transcriptome for AS analysis, especially under specific stress conditions [105].

Interestingly, our qPCR analysis revealed the upregulation of non-annotated transcripts in *SR30* (i.e., AS4-6), which also contain IR, suggesting the existence of more undetected AS or IR events. Additionally, our results showed that the transcript of *SR30* encoding the full-length protein, namely AS1, was induced by heat via changes in both transcription and AS. The distinctive upregulation of *SR30* among SRGs under heat stress suggest that it might play an important a role during plant heat responses, such as the maintaining of important AS events during splicing repression or regulation of heat-specific AS that is required during the heat acclimation process. In summary, our results provide evidence that the splicing regulators, especially *SR30*, themselves are regulated by dark and heat signals, which might, in turn, cause the changes in the global AS profile. In the future, it will be of research interest to identify the mRNA targets of these splicing regulators in plants under dark or high-temperature conditions using methods such as RNP immunoprecipitation (RIP) [106], which will provide more information on the splicing regulation network during plant responses to different abiotic signals.

## 4. Materials and Methods

### 4.1. Plant Materials and Treatment Conditions

*Arabidopsis thaliana* (Col-0) seeds were surface-sterilized with 20% bleach for 30 min, followed by three washes using sterile water, and were stratified at 4 °C in the dark for two days before germination on Petri dishes containing Murashige and Skoog (MS) medium [107] supplemented with 1.0% (*w*/*v*) agar and 1.5% (*w*/*v*) sucrose. Plants were grown at 22 °C under long-day conditions (16 h light/8 h dark photoperiod) with a light intensity of 100–150 μmol m^−2^s^−1^.

For transcriptome analysis, two-week-old seedlings were subjected to dark and heat treatments or kept unchanged under regular light conditions. For dark treatment, plates were wrapped with aluminum foil and were placed at their original place. For heat treatment, plates were placed at 39 °C in an incubator and were also covered by aluminum foil, which exposes the plants to homogeneous heat conditions in the dark [108]. The treatments started at around 10 am in the morning, when the plants had been exposed to light for approximately four hours. After 6 h, seedlings were collected and frozen immediately in liquid nitrogen and maintained at −80 °C until further use.

### 4.2. RNA Extraction and Transcriptome Analysis

Seedling samples were ground in liquid nitrogen and total RNA was extracted from an approximately 150 mg sample using the RNeasy Mini Kit (Qiagen, Hilden, Germany) following the manufacturer′s instructions. The integrity of extracted RNA was verified via electrophoresis on a 1.0% agarose gel. Short-read RNA sequencing and subsequent data analysis were conducted by Annoroad Gene Technology Co., Ltd. (Beijing, China) as previously described with minor modifications [109]. Briefly, a strand-specific library (~250 bp) was generated and the resulting cDNA library was used for sequencing on an Illumina HiSeq 4000 platform. Raw reads from 9 samples (three biological replicates for each condition) were trimmed to obtain clean reads for subsequent analysis. 

### 4.3. Analysis of RNA Sequencing Data

Arabidopsis reference genome annotation files were downloaded from TAIR. Mapping of clean reads and the analytical pipelines used for subsequent data analysis were as described previously [109]. Briefly, AS events were identified and quantified by using ASprofile (http://ccb.jhu.edu/software/ASprofile accessed on 1 May 2022). Significant changes in DEGs and DAS events were determined using log_2_FC > 2 and q-value (false discovery rate, FDR < 5%). The primarily identified DEGs and DAS events containing no expression value were filtered out. GO analysis was carried out by ShinyGo 0.76.3 (http://bioinformatics.sdstate.edu/go/ accessed on 1 May 2022) using the GO Biological Process database with an FDR cutoff of 0.05.

### 4.4. qPCR Analysis and RT-PCR Analysis

An amount of 2–4 μg of RNA was transcribed to cDNA with 1 μL oligo-dT primers in a 20 μL reaction volume using the First-Strand cDNA Synthesis Kit (Thermo Scientific, Rockford, IL, USA). For the RT-PCR assay, the resulting cDNA was diluted by about 10-fold and approximately 0.5 μL was used as a template for amplification in a 10 μL reaction volume using 2xTaq PCR StarMix (GenStar, Beijing, China). Amplified products were analyzed on a 1.0% agarose gel. DNA fragments were purified from the smear area in the PCR gel in Figure 6D by the E.Z.N.A.^®^ Gel Extraction Kit (Omega, Norcross, GA, USA) and were cloned using the A/Blunt-Zero Cloning Kit (Vazyme, Nanjing, China) for subsequent Sanger sequencing performed by BGI (Shenzhen, China). 

For qPCR analysis, 0.3 μL of cDNA was used as a template for amplification in a 10 μL reaction volume using the 2XTaq Pro Universal SYBR qPCR Master Mix (Vazyme, Nanjing, China) and the reactions were run on the CFX96/384 Real-Time Systems (Bio-Rad, Berkeley, CA, USA) with an initial denaturation at 95 °C for 1 min, followed by 40 cycles at 95 °C for 10 s, 58 °C for 10 s, and 72 °C for 10 s. Primers used for PCR analysis are listed in Table S2. Arabidopsis *ACT2* (AT3G18780) was used as the internal control. 

### 4.5. Subcellular Localization of SR30

Wide-type Arabidopsis was transformed with *Agrobacterium tumefaciens* strain GV3101 carrying *GFP-SR30* constructs by the floral dipping method. In addition, 8-day-old transgenic T2 seedlings grown on MS medium were covered by aluminum foil and were subjected to 39 °C for 6 h. Control plants were covered by aluminum foil and were put under normal growth conditions. After 6 h, seedling leaves were examined by a confocal microscope (Leica TCS SP8 imaging system).

## Data Availability

The RNA-seq data reported in this paper have been uploaded to the National Genomics Data Center (accession no. PRJNA904139).

## Supplementary Materials

The following supporting information can be downloaded at: https://www.mdpi.com/article/10.3390/ijms24065299/s1.
