# Supplementary material for: Global Analysis of Dark- and Heat-Regulated Alternative Splicing in Arabidopsis
Source: Int J Mol Sci. 2023 Mar 10;24(6):5299. doi: 10.3390/ijms24065299 (PMC10049525; doi:10.3390/ijms24065299)
Supplement: Supplementary file 1 [file ijms-24-05299-s001.zip › Supplementary Figures.pdf]

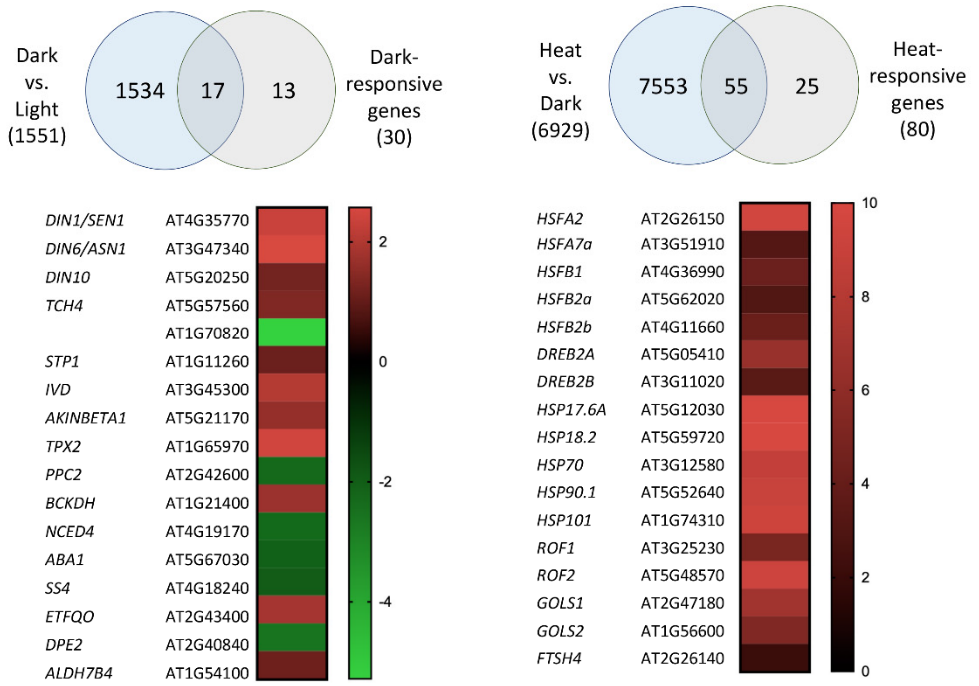

Figure S1. Comparison of previously reported dark- or heat- responsive genes with the dataset in this study.

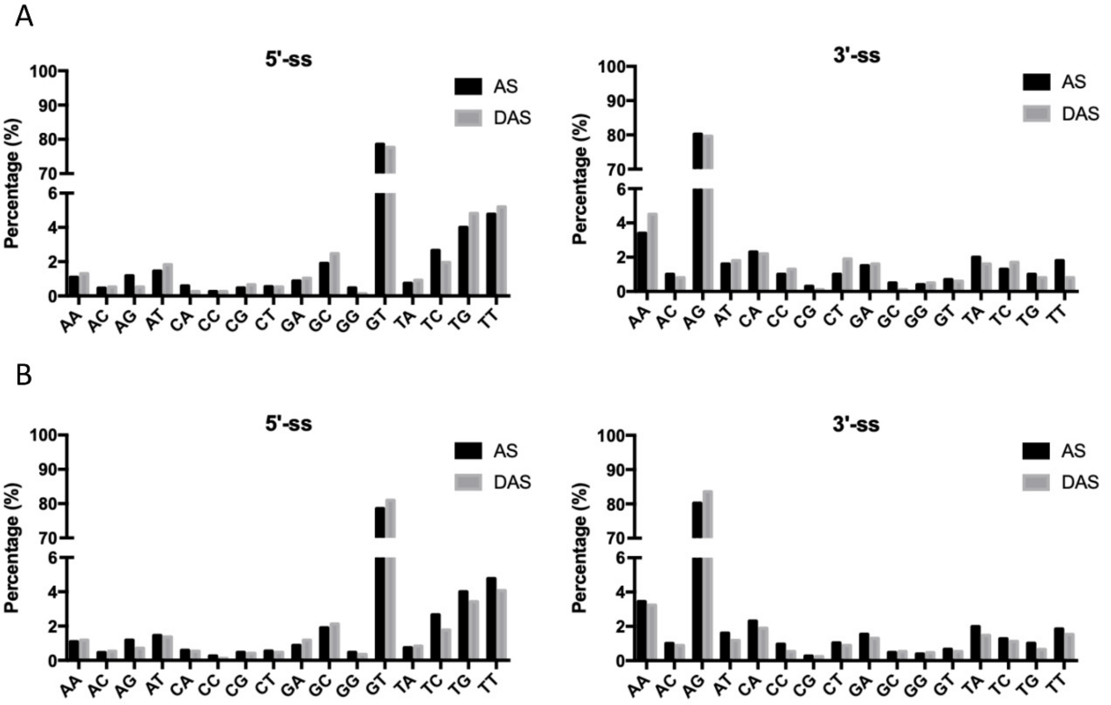

Figure S2. Analysis of the splice sites (ss) between total AS events and DAS events regulated by dark (A) and heat (B) treatments.

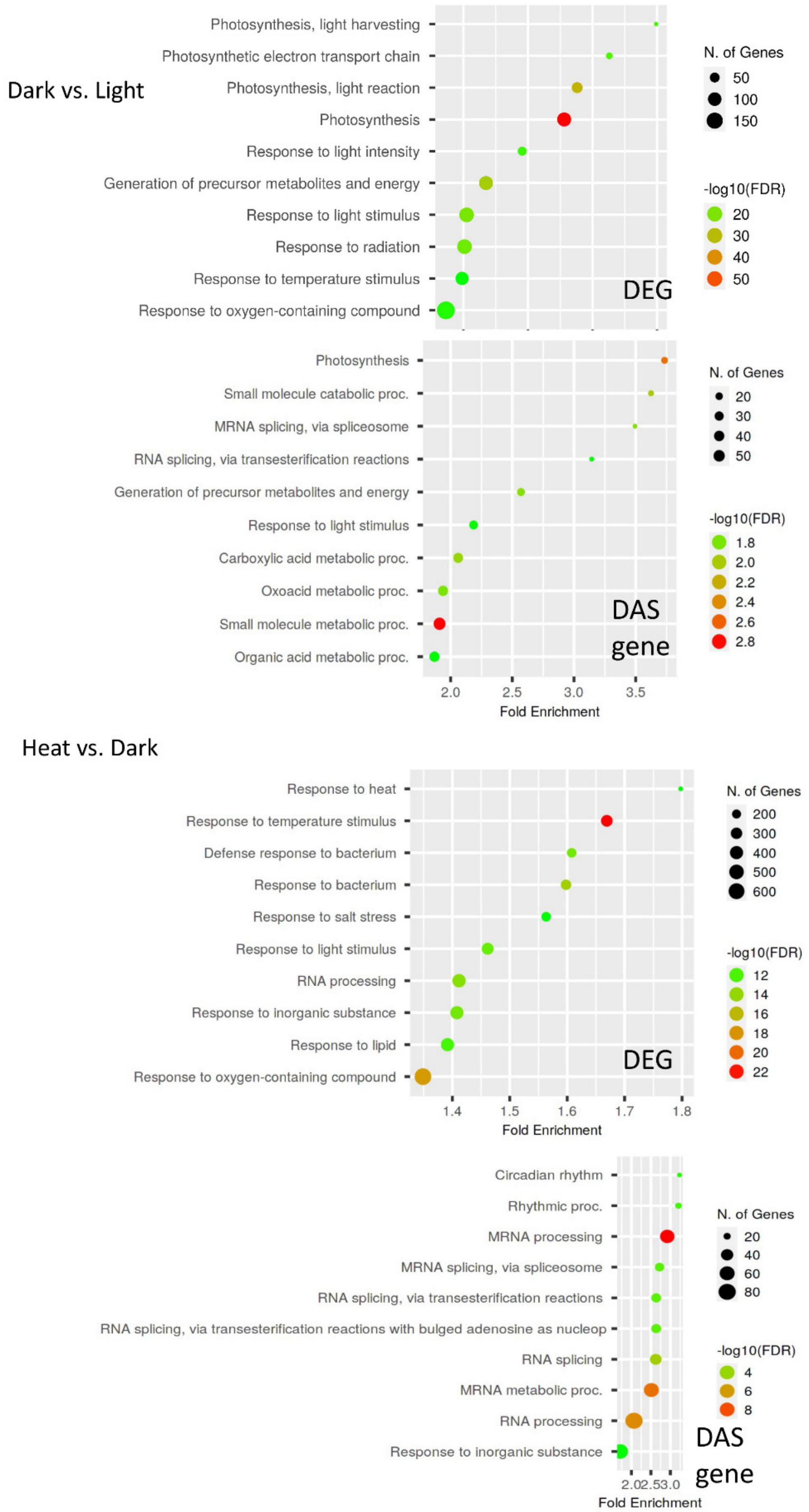

Figure S3. GO analysis of dark- or heat- regulated DEGs and DAS genes. Biological processes were sorted by fold enrichment and top ten pathways are shown.

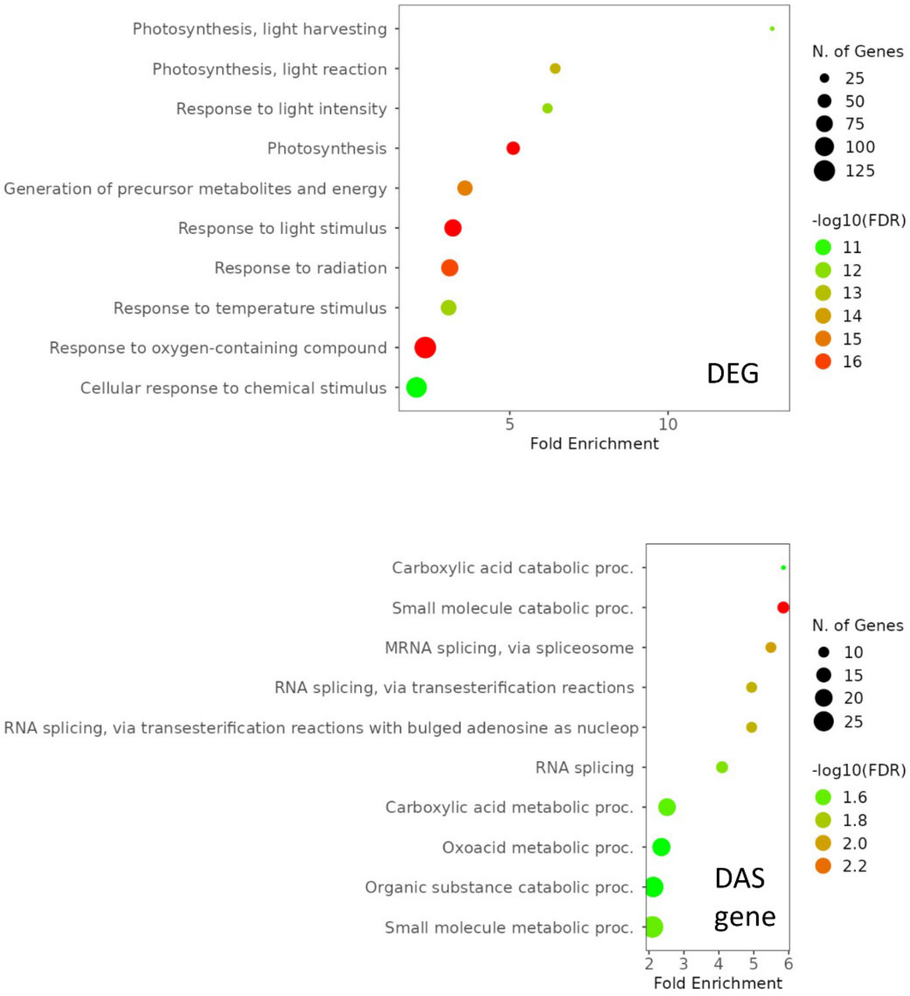

Figure S4. GO analysis of DEGs and DAS genes that were co-regulated by dark and heat treatments. Biological processes were sorted by fold enrichment and top ten pathways are shown.

AS5

ACACTGTGCTGCTTCATTCGTACGATGACGTACGTGAGAGACTCCACGAGCAGTAGTCAGACGACGAGCCCATCGCACCGCTTCCCGTG
GTGCGATCATAGCGGTGCAGATGTTCAGATGAAGCGGCATGAGCCACCAATATGTGGTACTCATCATGGCACGCAGCTTTCATTTATGC
TGCGCTCGTATGTTGTGGAATTGGAGCGGATACCATTTCACACAGGAAACATCTATGACCATGATTACGCCAAGCTCAGATTACCCTCAC
TAAAGGTACTAGTCCTGCAGGTTTAAACGAATTCGCCCTTCACCATGAGTAGCCGATGGAATCGTACGATCTACGTGGGATTTGCCTGG
AGATATTCGCAAGTGTGAGGTTGAAGATCTCTTCTACAAGTATGGACCAATTGTGGACATTGATTTGAAGATTCCACCGAGACCTCCTGG
TTATGCCTTTGTCGAGTTTGAAGATCCTCGTGATGCAGACGATGCAATTTATGGACGTGATGGTTATGATTTTGATGGGTGTCGACTTCG
GGTTAGTAAACGCATGATGAAAGCTAGCTTAATTTTCTGTAATTTCTTGTAAAGGTGTTATCTTTGTGTGATGTTTTTAGGTTGAGATTGC
ACATGGTGGTCGTAGATTTTCACCATCAGTTGATAGGTACAGCAGCAGCTACAGTGCGAGCCGTGCACCTTCAAGACGCTCTGACTACC
GCGTGCTTGTGACCGGATTACCGCCTTCTGCTTCGTGGCAGGACCTTAAGGATCACATGCGCAAAGCTGGAGATGTCTGCTTCTCTGAA
GTTTTCCCTGACCGTAAAGGCATGTCTGGGGTTGTGGATTATAGCAACTATGATGATATGAAGTACGCAATAAGGAAACTTGATGCCAC
TGAATTTCGAAATGCTTTCTCTAGTGCTTATATACGGGTGAGGGAATATGAGTCGAGGAGTGTGAGTCGAAGCCCAGATGATTCTAAAA
GCTATAGAAGCAGGAGTCGGAGCCGTGGTCCAAGCTGTAGCTATAGTAGCAAGAGCAGGAGTGTGTCACCTGCTAGATCCATTTCCCC
GCGTTCACGGCCCCTTAGTCGTTCTCGCTCGCTATACAGCTCTGTCTCAAGGTCCCAATCAAGATCAAAATCAAGATCAAGATCAAGATC
GAATTCTCCAGTTTCACCTGTGATATCTGGTTGAAAGGGCGAATTCGCGGCCGCTAAATCAATCGCCCTATAGAATGCCC

AS6

GGGCATCTATAGGGCGATTGATTTAGCGGCCGCGAATTCGCCCTTCACCATGAGTAGCCGATGGAATCGTACGATCTACGTTGGGAATT
TGCCTGGAGATATTCGCAAGTGTGAGGTTGAAGATCTCTTCTACAAGGTTTGAAAATTCCTCTTTTCTCTCGATAAAAATTGAATTCATTA
TGACTTGTTTGGGTTCATAAATTTGCAATTCTGTCTTGCTGAGACAATTTAAATCGACTCTTATGTATATTTGTTTCAGTATGGACCAATTG
TGGACATTGATTTGAAGATTCCACCGAGACCTCCTGGTTATGCCTTTGTCGAGTTTGAAGATCCTCGTGATGCAGACGATGCAATTTATG
GACGTGATGGTTATGATTTTGATGGGTGTCGACTTCGGGTTGAGATTGCACATGGTGGTCGTAGATTTTCACCATCAGTTGATAGGTAC
AGCAGCAGCTACAGTGCGAGCCGTGCACCTTCAAGACGCTCTGACTACCGCGTGCTTGTGACCGGATTACCGCCTTCTGCTTCGTGGCA
GGACCTTAAGGATCACATCCGCAAAGCTGGAGATGTCTGCTTCTCTGAAGTTTTCCCTGACCGTAAAGGCATGTCTGGGGTTGTGGATT
ATAGCAACTATGATGATATGAAGTACGCAATAAGGAAACTTGATGCCACTGAATTTCGAAATGCTTTCTCTAGTGCTTATATACGGGTGA
GGGAATATGAGTCGAGGAGTGTGAGTCGAAGCCCAGATGATTCTAAAAGCTATAGAAGCAGGAGTCGGAGCCGTGGTCCAAGCTGTA
GCTATAGTAGCAAGAGCAGGAGTGTGTCACCTGCTAGATCCATTTCCCCGCGTTCACGGCCCCTTAGTCGTTCTCGCTCGCTATACAGCT
CTGTCTCAAGGTCCCAATCAAGATCAAAATCAAGATCAAGATCAAGATCGAATTCTCCAGTTTCACCTGTGATATCTGGTTGAAAGGGCG
AATTCGTTTAACCTGCAGGACTAGTACCTTTATTGAGGGTTAATTCTGAGCTTGGCGTATCATGGCATAGCTGTTTCCTGTGTGAAATTGT
ATCCGCTCACATTCCCCCAACTACGGCCGGAGCAAAAAGTAAGCTGGGGGCTATGAGGGCTACCATATGGTGGGCTCCTGCCCCCTTCA
CTCGAACCTACTGCCCCGCTAAGATCGCACCCCCGCAGACGAGTGCGATTGGGGTC

Figure S5. Sequences of *SR30* isoforms identified by Sanger sequencing. Shaded sequences indicate those correspond to the seuqences of *SR30*. Underlined sequences indicate the retained intron sequence.
