# Supplementary material for: Global Analysis of Dark- and Heat-Regulated Alternative Splicing in Arabidopsis
Source: Int J Mol Sci. 2023 Mar 10;24(6):5299. doi: 10.3390/ijms24065299 (PMC10049525; doi:10.3390/ijms24065299)
Supplement: Supplementary file 1 [file ijms-24-05299-s001.zip › Supplementary Tables.pdf]

Supplementary Table S1. Summary of the basic parameters in short-read RNA sequencing dataset.

Sample	Clean reads	Unmapped reads	Mapped reads	Map rate
WT-CT-1	65944016	1589104	64354912	97.59%
WT-CT-2	48924508	1167567	47756941	97.61%
WT-CT-3	55942394	1317916	54624478	97.64%
WT-Dark-1	51500318	1275206	50225112	97.52%
WT-Dark-2	57206881	1512115	55694766	97.36%
WT-Dark-3	58275562	1508723	56766839	97.41%
WT-Heat-1	63452482	1613203	61839279	97.46%
WT-Heat-2	64318066	1744158	62573908	97.29%
WT-Heat-3	73779958	3929931	69850027	94.67%
Average	59927132	1739769	58187362	97.17%

Supplementary Table S2. Primer used for PCR analysis in this study.

Gene name	Gene ID	Primer name	Primer sequences
DIN1	AT4G35770	AS1 F	GAAAGCAACGACAACAAGCA
		AS1 R	TCTTAACCATTCCTGATCCGAC
		AS2 (IR) F	GAAAGCAACGACAACAAGCA
		AS2 (IR) R	GCAACGGTTTTGGCTTGG
DIN6	AT3G47340	AS1 F	CGACCCTGAATCCAAGATGATCAAG
		AS1 R	GCGTGATCTTTCAGGCCATC
		AS2 (IR) F	CACAAACTCAATACCTTCCTTCACA
		AS2 (IR) R	GCGTGATCTTTCAGGCCATC
DIN10	AT5G20250	AS1 F	TCAATTTTGGTCATACAAGAGAAGC
		AS1 R	ACCGCCGGTTTAATCGTCAT
		AS2 (IR) F	AACCCAGAATCAGACTTCATCTCA
		AS2 (IR) R	ACCGCCGGTTTAATCGTCAT
GT-1	AT1G13450	AS1 F	GAGACTCCTGGAAACGGTGATG
		AS1 R	AAGGGCATGTCTCGGTCAAG
		AS2 (IR) F	TGCTTGATTTATTCGTTCGGTCTC
		AS2 (IR) R	AAGGGCATGTCTCGGTCAAG
SPA3	AT3G15354	AS1 F	ATTTCATCAGCAGACCCGAC
		AS1 R	TGCTAACTCCCTGATTGATACT
		AS2 (IR) F	ATTTCATCAGCAGACCCGAC
		AS2 (IR) R	GTTTCAGTTTTCAATAGCCGACA
HYH	AT3G17609	AS1 F	CACAAGAAGCACAAAACTGAGGA
		AS1 R	GCGGCGTTTAGCTGTAGAGA
		AS2 (AE) F	ACAAGAAGCACAAAACTGCTGG
		AS2 (AE) R	GCGGCGTTTAGCTGTAGAGA
JMJD5	AT3G20810	Total F	GGTGGTGGGGAACTCCAATC
		Total R	GTGTAACGGAGTAACTGTCCCA
		AS2 (AE) F	GGTGGTGGGGAACTCCAATC
		AS2 (AE) R	CCAGCGAGAAGATCAGGCTGAGC
HSFA2	AT2G26150	Total F	GGAGGAACATGGGTTTGCAG
		Total R	CGAAACCGTATTGCCCAACC
		AS2 (CE) F	ATTCGTCAGCTCAATACTTATATGGG
		AS2 (CE) R	CGAAACCGTATTGCCCAACC
Luc7-lr	AT5G51410	AS1 F	TGCAAATTAATGAGCTATTCTAAG
		AS1 R	CTCAACGCTTTTGACCTCTT
		AS2 (IR) F	ATGTGATACCCCCTTTGGTT
		AS2 (IR) R	CTCAACGCTTTTGACCTCTT
RBM25	AT1G60200	AS1 F	CCATGCCTCCTCATGGGCTT
		AS1 R	GGGTCGTGGTGCTAGTTGAA
		AS2 (AE5) F	TGCTCTAGTTTGTGGGCTTC
		AS2 (AE5) R	GGGTCGTGGTGCTAGTTGAA

PRP4	AT2G41500	AS1 F	CTGGTCAGACTCAGAGGTTAT
		AS1 R	AATTGACGAAACCCGCGAAT
		AS2 (AE3) F	CTGGTCAGACTCAGAGGAAAC
		AS2 (AE3) R	AATTGACGAAACCCGCGAAT
SYF2	AT2G16860	AS1 F	TCCGCGAGATATCTGGTGTTC
		AS1 R	CGTAAGATTTTTCCCAAAACCTGT
		AS2 (AE3) F	TCCGCGAGATATCTGGTGTTC
		AS2 (AE3) R	ACTTCCAAGCGTTAAGAAACTGTG
U2AF65a	AT4G36690	AS1 F	GGTTATGTTACAGCCTGGT
		AS1 R	AAAGCACCAAACTTTCCGC
		AS2 (AE3) F	GGTTATGTTACAGCCTGGT
		AS2 (AE3) R	TCTTTATAGCAAAAGGCAAACT
RSZ32	AT3G53500	AS1 F	CTATGGAAACACTCGCCTCT
		AS1 R	CACATCTCGTACTCTTCCGTA
		AS2 (IR) F	CTATGGAAACACTCGCCTCT
		AS2 (IR) R	TATCCCCTGCACAACATCAA
RS40	AT4G25500	AS1 F	TGAAAGCTGGGTTTGCTTTT
		AS1 R	AATTCAAAGCGGTCAAGTGC
		AS2 (AE5&IR) F	TCAGGAAATACGGCAAGGTT
		AS2 (AE5&IR) R	GATAGTCCACCAGAGCCTTC
hnRNP-P	AT1G50300	AS1 F	ACCATTGGGTTGCTTAAGAGAGA
		AS1 R	CTCCCTTGGGCTCATCAGTT
		AS2 (AE5) F	GGTATGGTATTGGCCAGAGAGAC
		AS2 (AE5) R	CTCCCTTGGGCTCATCAGTT
SR30	AT1G09140	Total F	GATCGAATTCTCCAGTTTCACCT
		Total R	CGAGACAGAGTTTCCGGCTT
		AS1 F	TCTGTCTCAAGGTCCCAATCA
		AS1 R	CGAGACAGAGTTTCCGGCTT
		AS2 F	CTTAGTCGTTCTCGCTCGCT
		AS2 R	CCAGCTTTGTAAATCCAGTCC
		AS3 F	CTTAGTCGTTCTCGCTCGCT
		AS3 R	TCAACTGTGTACGGAAGCAA
		AS4 F	CGGGTATGTTGTATTGCTTTCTT
		AS4 R	ATCATCTGGGCTTCGACTCA
		AS5 F	GGTTAGTAAACGCATGATGAAAG
		AS5 R	AAATCTACGACCACCATGTGC
		CDS F	CACCATGAGTAGCCGATGGAATCGT
		CDS R	TCAACCAGATATCACAGGTGAA
ACT2	AT3G18780	F	TGTGCCAATCTACGAGGGT
		R	GCTGGTCTTTGAGGTTTCC
